# Exosome‐derived long non‐coding RNA AC010789.1 modified by FTO and hnRNPA2B1 accelerates growth of hair follicle stem cells against androgen alopecia by activating S100A8/Wnt/β‐catenin signalling

**DOI:** 10.1002/ctm2.70152

**Published:** 2025-01-02

**Authors:** Shaojun Chu, Lingling Jia, Yulong Li, Jiachao Xiong, Yulin Sun, Qin Zhou, Dexiang Du, Zihan Li, Xin Huang, Hua Jiang, Baojin Wu, Yufei Li

**Affiliations:** ^1^ Department of Plastic Surgery Shanghai East Hospital, School of Medicine, Tongji University Shanghai China; ^2^ Department of Military Medical Psychology Air Force Medical University Xi'an China; ^3^ St Hugh's College University of Oxford Oxford UK; ^4^ Department of Dermatology Hair Medical Center of Shanghai Tongji Hospital, Tongji Hospital, School of Medicine, Tongji University Shanghai China; ^5^ Department of Plastic Surgery Huashan Hospital Fudan University Shanghai China

**Keywords:** FTO, HFSCs, hnRNPA2B1, lncRNA AC010789.1, proliferation, S100A8

## Abstract

**Background:**

The increased incidence of androgenic alopecia (AGA) causes adverse physiological and psychological effects on people of all genders. The hair follicle stem cells (HFSCs) have displayed clinical improvements on AGA. However, the molecular mechanism of HFSCs against AGA remains elusive.

**Methods:**

The expression and prognosis of lncRNA AC010789.1 in AGA hair follicle tissues were assessed by qRT‐PCR analysis. CCK‐8, EdU and Transwell analysis were utilized to assess cell growth. The specific binding between AC010789.1 and FTO mediated m^6^A modification or the effect of AC010789.1 on hnRNPA2B1, S100A8 and Wnt/β‐catenin signaling expression was confirmed by bioinformatic analysis, RIP, RNA pull‐down and Western blot assay. The effects of Exosome‐loaded AC010789.1 prompted HFSCs proliferation and hair follicle regeneration were confirmed in hairless mice.

**Results:**

We herein found that the mRNA levels of lncRNA AC010789.1 were decreased in AGA tissue samples but increased in HFSCs of surrounding normal tissue samples. Overexpression (OE) of AC010789.1 promoted HFSC proliferation, DNA synthesis and migration as well as K6HF and Lgr5 upregulation, whereas knockdown of AC010789.1 showed the opposite effects. The total or AC010789.1 m^6^A levels were reduced and FTO demethylase was upregulated in AGA tissue samples, but these indicated the reverse results in HFSCs of surrounding normal tissue samples. FTO OE decreased AC010789.1 m^6^A levels and its mRNA levels in HFSCs and abolished AC010789.1‐induced HFSCs proliferation. In addition, AC010789.1 was identified to bind to m^6^A reader hnRNPA2B1, which was downregulated in AGA but upregulated in HFSCs of surrounding normal tissue samples. hnRNPA2B1 OE attenuated AC010789.1 knockdown‐induced inhibition of HFSCs proliferation. Moreover, AC010789.1 could bind to and enhance downstream S100A8 protein expression, which mediated Wnt/β‐catenin signaling to accelerate HFSCs proliferation. Exosome‐loaded AC010789.1 prompted HFSCs proliferation and hair follicle regeneration in mice.

**Conclusions:**

Our findings demonstrated that exosome‐derived lncRNA AC010789.1 modified by FTO and hnRNPA2B1 facilitated the proliferation of human HFSCs against AGA by activating S100A8/Wnt/β‐catenin signaling.

**Key points:**

- Long non‐coding RNA (lncRNA) AC010789.1 was downregulated in hair follicle tissues from androgenic alopecia (AGA) and upregulated in hair follicle stem cells (HFSCs).
- LncRNA AC010789.1 promoted the proliferation and migration of HFSCs.
- FTO/hnRNPA2B1‐mediated m^6^A modification of lncRNA AC010789.1 promoted HFSCs growth by activating S100A8/Wnt/β‐catenin signalling.
- Exosome‐derived AC010789.1 accelerated HFSCs proliferation.

## Graphical abstract

1. Long non‐coding RNA (lncRNA) AC010789.1 was downregulated in hair follicle tissues from androgenic alopecia (AGA) and upregulated in hair follicle stem cells (HFSCs).
2. LncRNA AC010789.1 promoted the proliferation and migration of HFSCs.
3. FTO/hnRNPA2B1‐mediated m^6^A modification of lncRNA AC010789.1 promoted HFSCs growth by activating S100A8/Wnt/β‐catenin signalling.
4. Exosome‐derived AC010789.1 accelerated HFSCs proliferation.

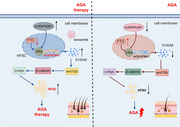

## 1 INTRODUCTION

Androgenetic alopecia (AGA) is the leading reason for hair thinning in men which contributes to persons’ poor quality of life. The pathogenesis of AGA is associated with genetic susceptibility and increased dihydrotestosterone (DHT) in androgen‐sensitive hair follicles.^1^ Medical treatments such as minoxidil and oral finasteride, as nonmedical treatments including platelet‐rich plasma injection and hair transplant have been applied for the treatment of AGA.^2^ Additionally, The destiny of HFSCs be controlled by Wnt/β‐catenin, Notch and BMP signalling pathways^3^, ^4^ and HFSCs‐based tissue engineering may provide promising therapeutic strategies for AGA.^5^, ^6^

It is known that dysregulation of long non‐coding RNA (lncRNA) is implicated in the disease process of AGA. LncRNA, microRNA and messenger RNA (mRNA) can interact to create a competitive endogenous RNA network that has an impact on the hair follicle cycle^7^ and AGA.^8^ Dermal papilla cells (DPCs) activate the regrowth of hair follicles. It has been shown that lncRNA XIST, lncRNA H19 or lncRNA PCAT1 facilitates DPCs‐induced hair follicle regeneration by activating the hedgehog or Wnt signalling,^9^, ^10^, ^11^ while lncRNA AL136131.3 represses PPARγ‐mediated hair proliferation in AGA.^12^ We previously identified lncRNA AC010789.1 deregulation in AGA and HFSCs,^13^ but the role of lncRNA AC010789.1 in HFSCs regeneration against AGA remains unknown.

N6‐methyladenosine (m^6^A) is recognized for the predominant alteration in eukaryotic mRNAs and functions depending on the m^6^A methyltransferases (such as METTL3, etc), demethylases (FTO and ALKBH5) and m^6^A readers (YTHDF1‐3, etc).^14^ Deregulation of m^6^A components is essential to the development of skin diseases including skin cancer.^15^, ^16^ METTL3 promotes proper epithelial self‐renewal^17^ and oocyte and follicle development,^18^ while FTO represses hair follicle‐related neural crest stem cell differentiation.^19^ The lncRNA AGAP2‐AS1, which is modified by m6A, promotes the development of psoriasis through the miR‐424‐5p/AKT3 signalling pathway.^20^ Our previous study showed that the m6A alteration of lncRNA SNHG3, catalyzed by METTL3, boosts the growth and invasiveness of melanoma.^21^ However, m^6^A modification of lncRNA AC010789.1 in HFSCs against AGA remains unreported.

It has been shown that PlncRNA‐1, modulates the growth and individualization of HFSCs via the Wnt/β‐catenin signalling pathway.^22^ Our current investigation indicates that lncRNA AC010789.1 was suppressed in AGA but upregulated in HFSCs; FTO/hnRNPA2B1‐mediated m^6^A modification of AC010789.1 enhanced the growth of HFSCs stimulation of S100A8/Wnt/β‐catenin signalling; Exosome‐loaded AC010789.1 accelerated HFSCs proliferation and hair follicle restoration in mice. Our findings might provide a promising therapeutic strategy for AGA persons.

## 2 MATERIALS AND METHODS

### 2.1 Clinical samples

Ten cases of hair follicle tissue specimens derived from persons with AGA were provided by Shanghai East Hospital and saved Stored in liquid nitrogen at ‐80°C for real‐time quantitative polymerase chain reaction (qRT‐PCR) and Western blot analysis.

### 2.2 HFSCs isolation and culture

The HFSCs isolation and culture was performed according to our previous report.^13^

### 2.3 RNA extraction and qRT‐PCR

Extract total RNA with Trizol (74104; QIAGEN). Then, the RNA was translated into cDNA through a reverse transcription reaction following the directions of the reverse transcription kit (R233; VAZYME). The cDNA preparations were analyzed qRT‐PCR with the SYBR Master Mix kit (Q111‐02; VAZYME). The reaction system was composed of SYBR Mix (10 µL), forward primer (0.4 µL), reverse primer (0.4 µL), 50× ROX Reference Dye 2 (0.4 µL), and H2O (4.8 µL). RT‐PCR System(Viia7; ABI) instruments were utilized in combination with reaction conditions of 95°C (10 min), 40 cycles of 95°C (15 s) and 60°C (60s). The cycle threshold (Ct) for each well was documented, with triplicate measurements taken for every sample. The relative quantification of the product's expression was estimated with b‐actin as the internal reference using the 2^−ΔΔCt^ method.

### 2.4 Western blot analysis

AGA hair follicle tissues were pulverized using liquid nitrogen and then lysed by RIPA lysis buffer (G2002; Servicebio) which contained 1  mM phenylmethylsulfonyl fluoride and 1  mM sodium orthovanadate (S1873; Beyotime). HFSCs and AGA hair follicle tissues were dissolved and set on ice(30 min) for centrifugation (4°C, 12 000 rpm and 10 min). Then, a 1.5  mL centrifuge tube was employed to draw off the supernatant to store at −80°C. Following the denaturation of the protein by mixing it with 5× loading buffer and boiling in a water bath, the proteins were subjected to electrophoresis using electrodes (80 and 120 V). Next, the protein was transferred to PVDF membranes (0.22/0.45 µm, IPVH00010; Millipore) by wet transfer method for 90 min‐135 min. Subsequently, the membranes were incubated in a TBST sealing solution with 5% skimmed milk at ambient temperature (2 h). The membrane was kept incubating with the primary antibody overnight, targeting FTO (1:3000, 27226‐1‐AP; Proteintech), hnRNPA2B1 (1:5000, 67445‐1‐Ig; Proteintech), K6HF (1:1000, DF9009; Affinity), Lgr5 (1:1000, ab75850; Abcam), S100A8 (1:1000, DF6556; Affinity), Wnt10b (1:1000, DF9038; Affinity), c‐myc (1:1000, AF6054; Affinity) and GAPDH (1:1000, AB‐P‐R001; GOODHERE BIOTECH) on a shaker(4°C). After being washed with TBST for 5–6 rounds, each lasting 5 min, the membranes were then incubated with a secondary antibody conjugated to horseradish peroxidase (HRP) (2 h, 37°C), followed by additional TBST washings for 5 min each. Next, the membranes were subjected to a mixture comprising the enhancer solution in ECL reagent(G2014; Servicebio)with stable peroxidase solution (1:1) (min). Once the protein bands became clearly visible, the surplus substrate solution was blotted away using filter paper, after which the membranes were fixed and the colour development process was initiated.

### 2.5 Plasmid, short hairpin RNA and cell transfection

The lncRNA AC010789.1 overexpression (OE) lentiviruses, small‐interfering RNA (siRNA) targeting AC010789.1 (si‐AC010789.1), the FTO OE plasmids, the hnRNPA2B1 OE plasmids and siRNA targeting S100A8 (si‐S100A8) were acquired from GenePharma. Negative control (NC), Vector and si‐NC were regarded as control groups. HFSCs were seeded in 6‐well plates (24 h) before transfection of si‐AC010789.1, AC010789.1 lentiviruses, FTO plasmids, hnRNPA2B1 plasmids or si‐S100A8 with 50%–60% confluence, and Subsequently, Lipofectamine 2000 (Invitrogen) was used to facilitate the transfection, following the manufacturer's guidelines.

### 2.6 Flow cytometry

1 × 10^6^ HFSCs were collected in a tube containing 0.1 mL of phosphate‐buffered saline (PBS) with 0.5% bovine serum albumin (BSA) and combined with PE anti‐human CD29 antibody (303003, BioLegend) and FITC anti‐human CD71 antibody (334103, BioLegend) according to the requirements at 4°C in dark for 30 min. After resuspension with 0.2 mL PBS added 0.5% BSA, flow cytometry analysis was performed.

### 2.7 Cell Counting Kit‐8 and Transwell assays

The procedures for these assays were as detailed in our previous studies.^12^, ^13^, ^21^

### 2.8 5‐ethynyl‐2'‐deoxyuridine assay

5‐ethynyl‐2'‐deoxyuridine assay (EdU, C0071s; Beyotime) was used to label the HFSCs, which were added with 1 mL of 4% paraformaldehyde after the culture medium was removed. Then, the cells were immobilized at ambient temperature for a duration of 15 min. Following the cell wash, 0.5 mL of the Click reaction mixture was inserted into each well, and the plates were incubated in the dark (30 min). Subsequent to the cell washing, they were then added with 1 mL of 1X Hoechst 33342 solution (C1025; Beyotime) to each well and incubated in the dark (10 min). The products were washed and sealed with a sealing solution, then we collected the images under a fluorescence microscope.

### 2.9 RNA immunoprecipitation and m^6^A RIP‐PCR

The RNA‐binding protein immunoprecipitation kit (17‐700; Millipore) and anti‐m^6^A (68055‐1‐Ig; Proteintech), anti‐FTO (27226‐1‐AP; Proteintech), anti‐S100A8 (DF6556; Affinity) and anti‐ hnRNPA2B1 (67445‐1‐Ig; Proteintech) were applied for RNA immunoprecipitation (RIP) and m^6^A RIP‐PCR (MeRIP‐PCR) assays following the instructions specified by the manufacturer.

### 2.10 RNA pull‐down

RNA‐binding Protein Immunoprecipitation Kit (17‐700; Millipore) was conducted to accomplish this experiment. To label the RNA probe Biotin and bind it to Pierce Streptavidin Magnetic Beads (88816; Thermo), 50 µL magnetic beads were added into a 1.5 mL EP tube, and placed in a magnetic rack for 5 min. Then, we collected magnetic beads and discarded the supernatant. Resuspend magnetic beads, then blend with a 20 mM Tris (equal volume, PH7.5). Then, an RNA Capture Buffer of equal volume to resuspend magnetic beads was added, and 50 pmol labelled RNA was added. For RNA protein binding, an equivalent measure of 20 mM Tris (PH7.5) magnetic beads were reconstituted. 100 µL Protein RNA Binding Buffer was mixed (4°C, 30–60 min). The above liquid was placed in a magnetic rack for 5 min, and magnetic beads were gathered. Remove the supernatant to a fresh centrifuge tube to serve as a control sample. 100 µL wash buffer and 50 µL Elution buffer were incorporated into the magnetic beads and incubated at 37°C for 15–30 min. They were precipitated in a magnetic frame for 5 min and RNA binding protein mixtures obtained were used for Western blot or mass spectrum analysis.

### 2.11 Silver impregnation and mass spectrum analysis

Note that, 20 µL RNA binding protein mixtures from RNA pull‐down assay were loaded onto 10% sodium dodecyl sulfate‐polyacrylamide gel electrophoresis (SDS‐PAGE). The silver staining procedure was performed following the guides of the silver staining kit (C500029; Sangon) after electrophoresis. The polyacrylamide gel was taken out after electrophoresis and washed with distilled water. We took a photo of the silver‐stained SDS‐PAGE, cut the different protein bands between ENST0000004589.1 and the NC lane and sent them to the mass spectrometry platform for protein identification.

### 2.12 Establishment of exosome‐derived lncRNA AC010789.1

Adipose‐derived stem cells (ADSCs) in vitro were transfected with lncRNA AC010789.1 OE lentiviruses and the control group. The ADSCs were placed in a 37°C, 5% CO_2_ incubator for culture. The exosomes (Exo‐AC010789.1 and Exo‐NC) were extracted using ultracentrifugation (4°C, 12 000 × RPM). qRT‐PCR was employed for the detection of the expression of lncRNA AC010789.1 in exosomes. The stability was assessed by qRT‐PCR after treatment with actinomycin D. The electron microscopy was utilized to examine the morphology of extracellular vesicles. NTA was used to measure the size distribution of extracellular vesicles, while Western blot analysis was applied to determine the presence of extracellular vesicle markers (ALIX and Calnexin).

### 2.13 In vivo tumorigenesis model

Shanghai Laboratory Animal Center supplied the 7‐week‐old male mice. We used hair removal cream to prepare a hairless mouse model on the back. 5% Minoxidil was used as a positive control. 200 µL PBS containing 2.0 × 10^9^ Exo‐AC010789.1 or Exo‐NC was subcutaneously injected into 10 spots on the dorsal skin (20 µL in each spot). The hair coverage ratio was detected after injection into the back of mice at 0, 10th, and 20th days. Then the mice were executed, and their skin tissues were sliced. The morphology of the skin tissue was analyzed using Hematoxylin and Eosin (HE). Immunohistochemistry (IHC) and immunofluorescence (IF) were used for the analysis of S100A8, Wnt10b, β‐catenin, c‐myc, K6HF and Lgr5 protein expression in the skin tissues according to the previous report.^12^

### 2.14 Statistical analysis

GraphPad Prism 5 software was utilized to conduct statistical analyses. Data are shown as the mean ± standard deviation. For group comparisons, chi‐square tests, student's t‐tests and analysis of variance (ANOVA) were employed.

## 3 RESULTS

### 3.1 LncRNA AC010789.1 was downregulated in hair follicle tissues from AGA and upregulated in HFSCs

**FIGURE 1:**
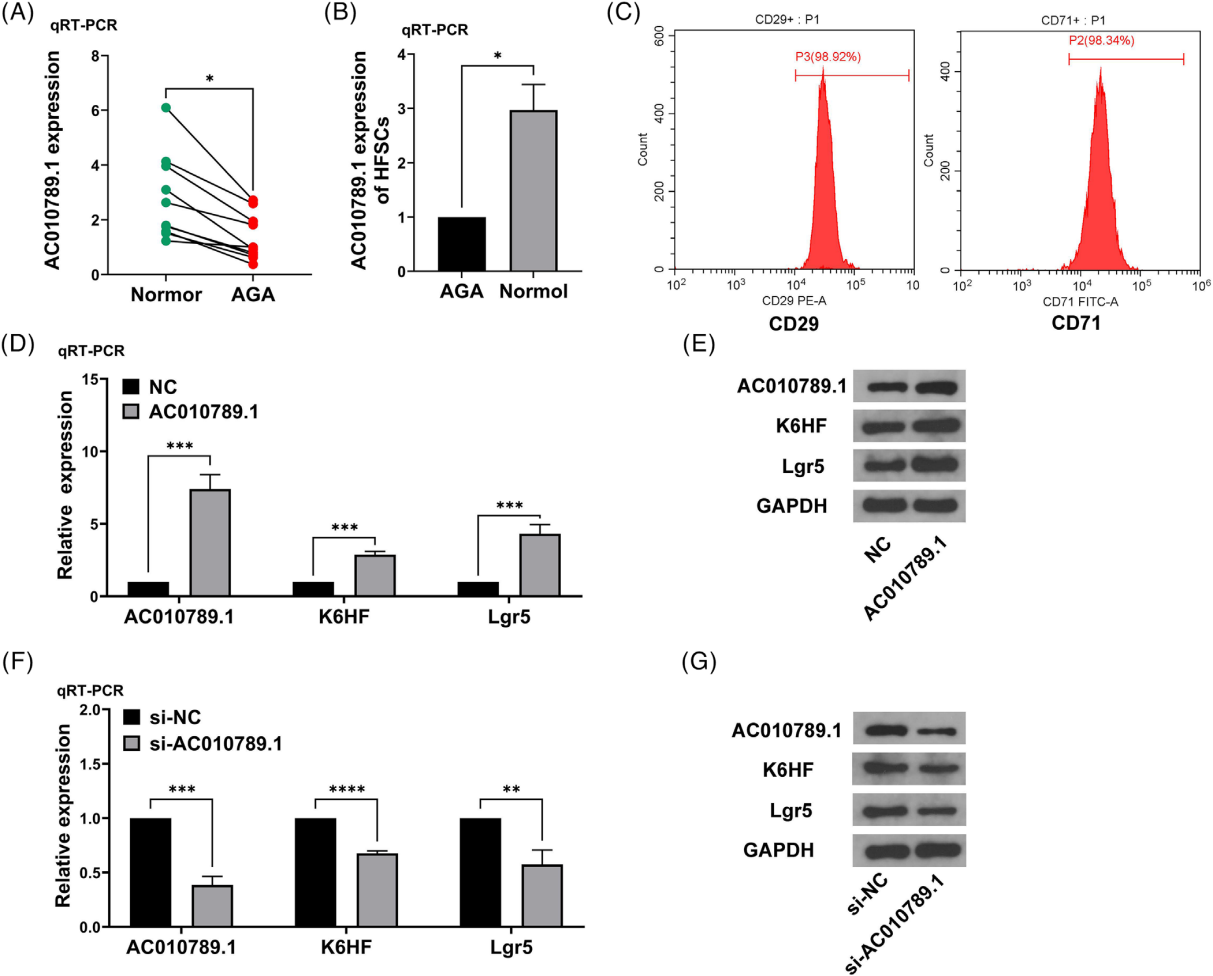
Long non‐coding RNA (lncRNA) AC010789.1 was downregulated in hair follicle tissues from androgenic alopecia (AGA) and upregulated in hair follicle stem cells (HFSCs). (A) Real‐time quantitative polymerase chain reaction (qRT‐PCR) analysis of the expression levels of AC010789.1 in 10 pairs of AGA hair follicle tissues. (B) qRT‐PCR analysis of the expression levels of AC010789.1 in HFSCs. (C) 5‐ethynyl‐2'‐deoxyuridine assay (EdU) flow cytometry analysis of the molecular markers CD29 and CD71 of HFSCs. (D) qRT‐PCR analysis of the expression levels of AC010789.1 after the transfection with AC010789.1 OE lentiviruses into HFSCs and K6HF and Lgr5 after the transfection with AC010789.1 OE lentiviruses into HFSCs. (E) Western blot analysis of the expression levels of AC010789.1, K6HF and Lgr5 after the transfection with AC010789.1 OE lentiviruses into HFSCs. (F) qRT‐PCR analysis of the expression levels of AC010789.1 after transfection with si‐AC010789.1 into HFSCs and K6HF and Lgr5 after transfection with si‐AC010789.1 into HFSCs. (G) Western blot analysis of the expression levels of AC010789.1, K6HF and Lgr5 after transfection with si‐AC010789.1 into HFSCs. Data shown are the mean ± SEM of three experiments. *p < .05, **p < .01 and ***p < .001.

We previously identified the differentially expressed lncRNA AC010789.1 between hair follicle tissues from AGA and healthy persons.^13^ qRT‐PCR analysis was used to determine the expression levels of lncRNA AC010789.1 in 10 pairs of hair follicle tissues from AGA, indicating that the mRNA expression of AC010789.1 was reduced in AGA hair follicle as in comparison with those in normal hair follicle tissues (Figure 1A). We isolated the HFSCs from the AGA hair follicle tissues^13^ but found that the mRNA levels of AC010789.1 were increased in HFSCs of surrounding normal tissue samples as compared with the HFSCs of from the AGA hair follicle tissues (Figure 1B). Primary HFSCs were verified through the detection of the molecular markers CD29 and CD71 by flow cytometry (Figure 1C). Then, qRT‐PCR was used to test HFSCs that were transfected with AC010789.1 OE lentiviruses and the transfection efficiency (Figure 1D). qRT‐PCR and Western blot analyses showed that AC010789.1 OE raised the mRNA and protein expressions of K6HF and Lgr5 in HFSCs (Figure 1E,F and Figure S1A), while AC010789.1 knockdown (KD) lowered their levels in HFSCs than the control group (Figure 1G–I and Figure S1B).

### 3.2 LncRNA AC010789.1 promoted the proliferation and migration of HFSCs

**FIGURE 2:**
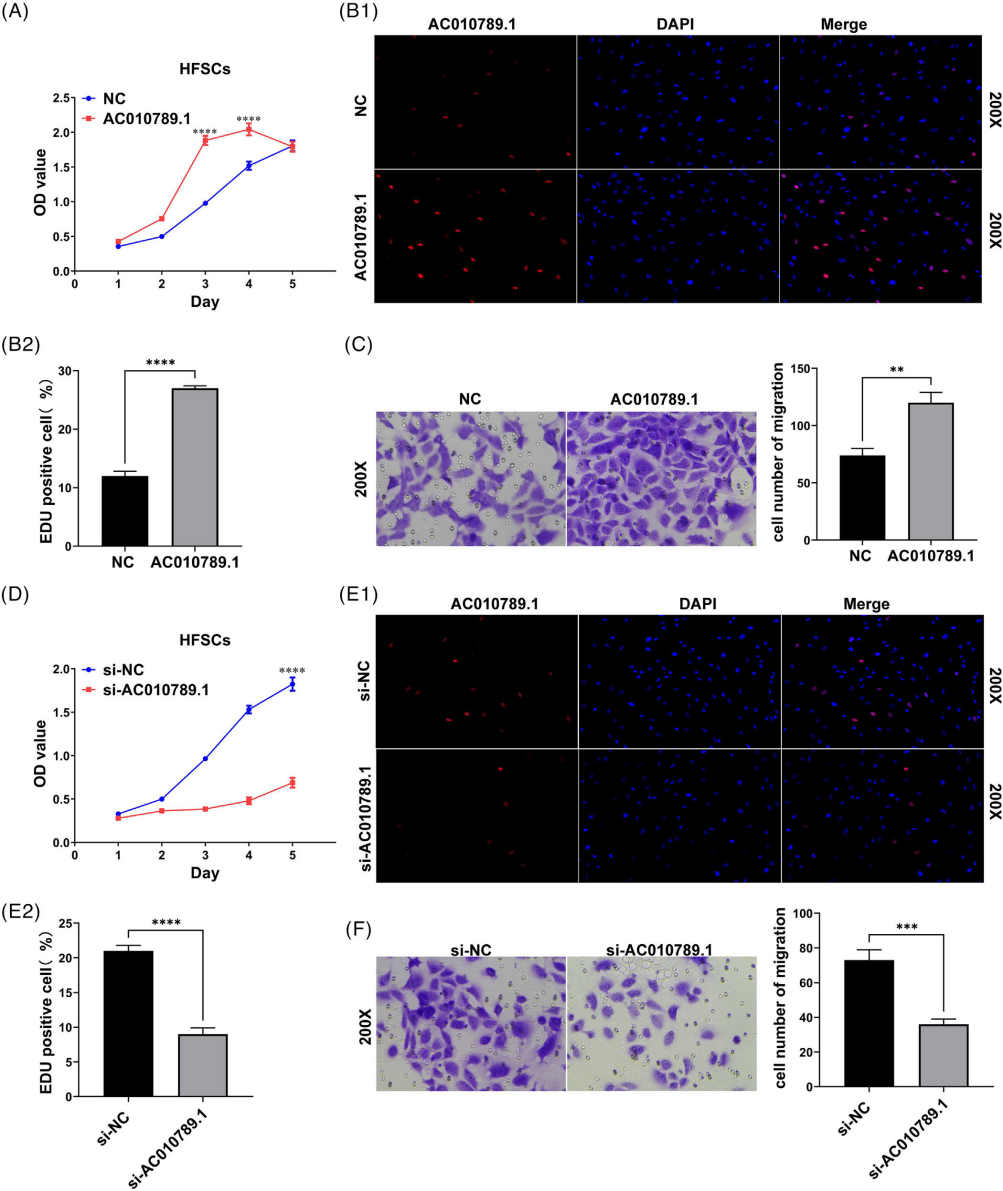
LncRNA AC010789.1 promoted the proliferation and migration of hair follicle stem cells (HFSCs) (A) CCK8 analysis of the cell proliferation viability after the transfection with AC010789.1 OE lentiviruses into HFSCs. (B) 5‐ethynyl‐2'‐deoxyuridine assay (EdU) assays of the DNA synthesis after the transfection with AC010789.1 OE lentiviruses into HFSCs. (C) Transwell analysis of cell migration capabilities after the transfection with AC010789.1 OE lentiviruses into HFSCs. (D) CCK8 analysis of the cell proliferation viability after the transfection with si‐AC010789.1 into HFSCs. (E) EdU assays of the DNA synthesis after the transfection with si‐AC010789.1 into HFSCs. (F) Transwell analysis of cell migration capabilities after the transfection with si‐AC010789.1 into HFSCs. Data shown are the mean ± SEM of three experiments. **p < .01, ***p < .001 and ****p < .0001.

To elucidate the role of lncRNA AC010789.1 in HFSCs, Cell Counting Kit‐8 (CCK‐8) and EdU assays suggested that the cell growth and DNA synthesis were up‐regulated by AC010789.1 OE in HFSCs (Figure 2A,B). Transwell assay showed that the migration capabilities of HFSCs were augmented by AC010789.1 OE (Figure 2C). Conversely, the proliferation and DNA synthesis were decreased by AC010789.1 KD in HFSCs (Figure 2D, E). The Transwell assay showed that the migration abilities of HFSCs were weakened by AC010789.1 KD (Figure 2F).

### 3.3 FTO mediated m^6^A modification of lncRNA AC010789.1 in HFSCs

**FIGURE 3:**
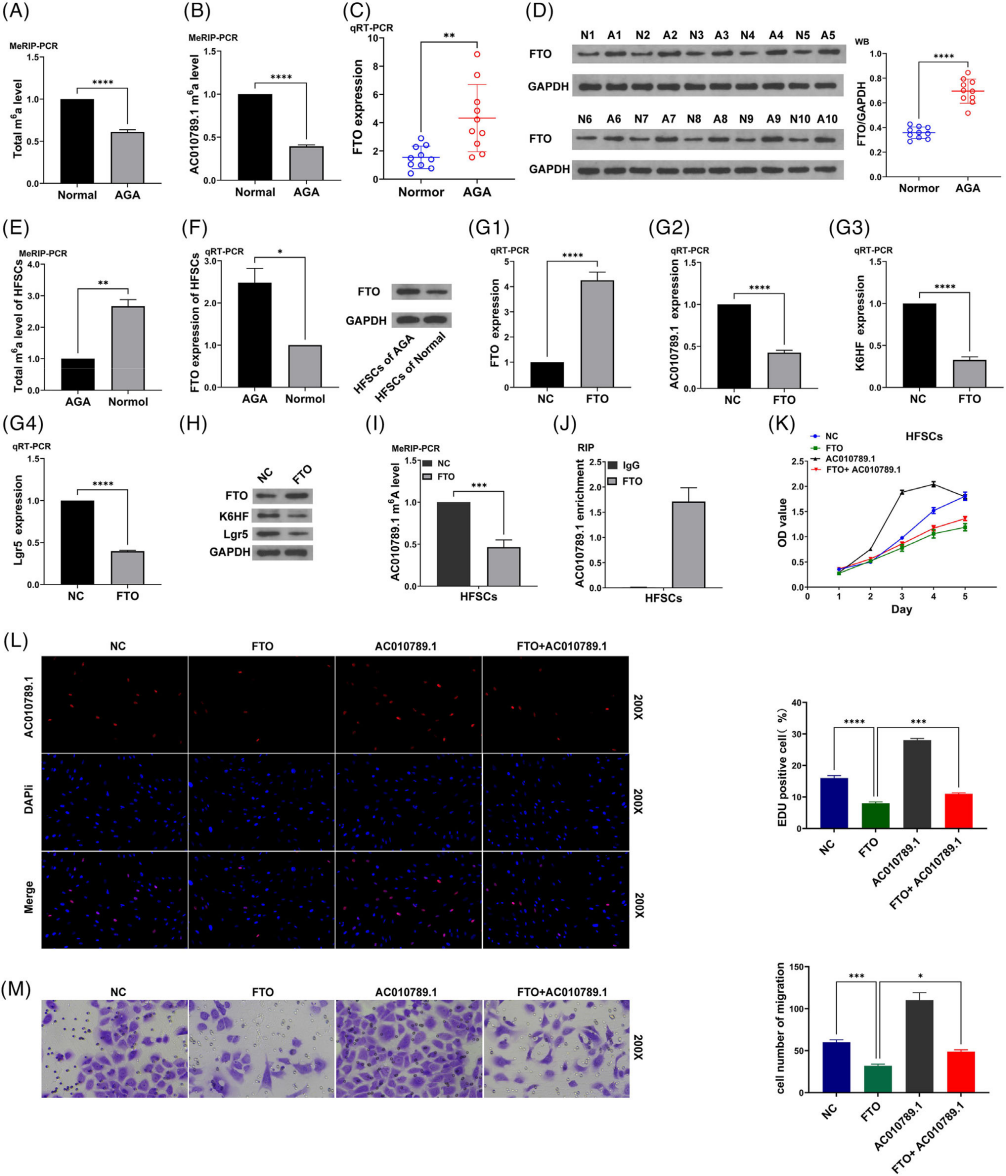
FTO mediated m^6^A modification of long non‐coding RNA (lncRNA) AC010789.1 in hair follicle stem cells (HFSCs) (A, B) MeRIP‐PCR analysis of the total m^6^A levels and AC010789.1 m^6^A levels in androgenic alopecia (AGA) hair follicle tissues. (C, D) Real‐time quantitative polymerase chain reaction (qRT‐PCR) and Western blot analysis of the expression levels of FTO in AGA hair follicle tissues. (E) MeRIP‐PCR analysis of the total m^6^A levels in AGA HFSCs. (F) qRT‐PCR and Western blot analysis of the expression levels of FTO in HFSCs. (G, H) qRT‐PCR and Western blot analysis of the expression levels of FTO, AC010789.1, K6HF and Lgr5 after the transfection with FTO OE plasmids into HFSCs. (I) MeRIP‐PCR analysis of the effects of FTO OE on AC010789.1 m^6^A levels in HFSCs. (J) RIP analysis of the endogenous interaction of AC010789.1 mRNAs with FTO protein in HFSCs. (K‐M) CCK‐8, 5‐ethynyl‐2'‐deoxyuridine assay (EdU) and Transwell analysis of the cell proliferation and migration viability after the co‐transfection with FTO and AC010789.1 OE lentiviruses into HFSCs. Data shown are the mean ± SEM of three experiments. *p < .05, **p < .01, ***p < .001 and ****p < .0001.

We predicted that m^6^A sites can be enriched in lncRNA AC010789.1 (Figure S2A). MeRIP‐PCR analysis showed that the total m^6^A levels or AC010789.1 m^6^A levels were found reduced in AGA hair follicle tissues as compared with those in normal hair follicle tissues (Figure 3A,B). We hypothesized that FTO as an m^6^A demethylase may be involved in the m^6^A modification of lncRNA AC010789.1. The mRNA and protein expressions(qRT‐PCR and Western blot analyses) of FTO were markedly elevated in 10 pairs of AGA hair follicle tissues relative to the normal hair follicle tissues (Figure 3C,D). In addition, the total m^6^A levels were found increased in HFSCs from the normal hair follicle tissues (Figure 3E), while the mRNA and protein expressions of FTO were decreased in HFSCs as compared with HFSCs from the AGA hair follicle tissues (Figure 3F and Figure S2B). FTO OE inhibited the mRNA levels of AC010789.1 as well as the mRNA and protein expressions of K6HF and Lgr5 in HFSCs (Figure 3G,H and Figure S2C), but we found that ALKBH5 or METTL3 OE exhibited no impact on the expression of lncRNA AC010789.1 in HFSCs (Figure S2D–G). FTO OE reduced the m^6^A levels of lncRNA AC010789.1 in HFSCs (Figure 3I), and AC010789.1 mRNAs could interact with and be enriched in FTO protein in HFSCs (Figure 3J). Functionally, FTO OE suppressed the proliferation, DNA synthesis and migration of HFSCs and AC010789.1 OE abolished FTO‐induced suppressive effects on the cell growth, DNA synthesis and migration of HFSCs (Figure 3K–M).

### 3.4 hnRNPA2B1 interacted with lncRNA AC010789.1 to accelerate HFSCs growth

**FIGURE 4:**
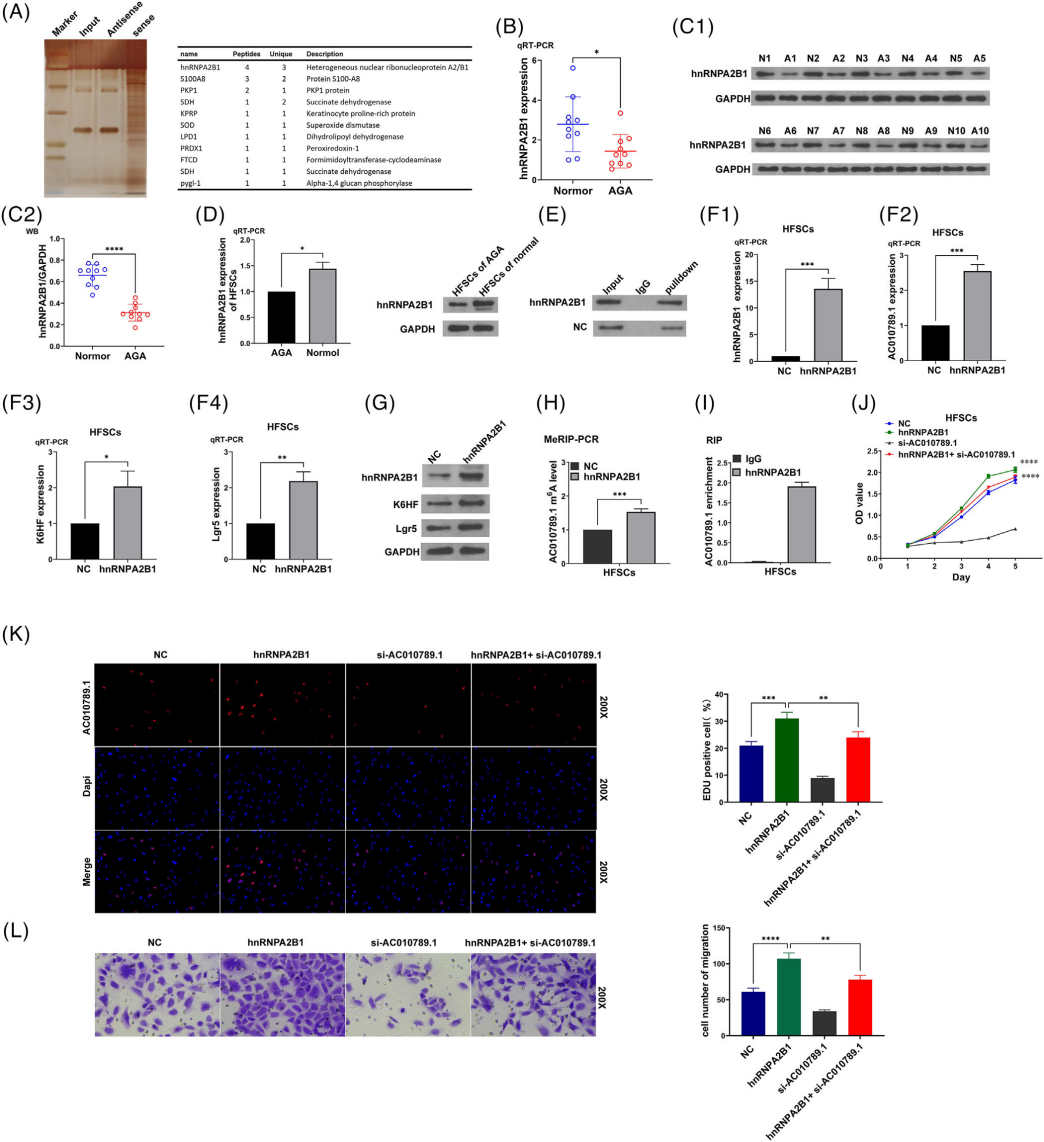
hnRNPA2B1 interacted with long non‐coding RNA (lncRNA) AC010789.1 to promote hair follicle stem cells (HFSCs) growth. (A) Silver impregnation and Mass spectrum analysis of AC010789.1‐interacting proteins in HFSCs. (B, C) Real‐time quantitative polymerase chain reaction (qRT‐PCR) analysis of the expression levels of hnRNPA2B1 in 10 pairs of androgenic alopecia (AGA) hair follicle tissues. (D) qRT‐PCR and Western blot analysis of the expression levels of hnRNPA2B1 in HFSCs. (E) RNA pull‐down verification of the interaction of hnRNPA2B1 protein with the AC010789.1 mRNAs in HFSCs. (F, G) qRT‐PCR and Western blot analysis of the expression levels of hnRNPA2B1, AC010789.1, K6HF and Lgr5 after the transfection with hnRNPA2B1 OE plasmids into HFSCs. (H) MeRIP‐PCR analysis of the effects of hnRNPA2B1 OE on AC010789.1 m^6^A levels in HFSCs. (I) RIP analysis of the enrichment of the endogenous AC010789.1 in hnRNPA2B1 protein in HFSCs. (K‐M) CCK8, 5‐ethynyl‐2'‐deoxyuridine assay (EdU) and Transwell analysis of the cell proliferation and migration viability after the co‐transfection with hnRNPA2B1 OE plasmids and si‐AC010789.1 into HFSCs. Data shown are the mean ± SEM of three experiments. *p < .05, **p < .01, ***p < .001 and ****p < .0001.

We further sought AC010789.1‐interacting proteins in HFSCs by Mass Spectrum and identified m^6^A reader hnRNPA2B1 could bind with AC010789.1 in HFSCs (Figure 4A). Analyses conducted through quantitative real‐time PCR and Western blot revealed that the expression levels of mRNA and protein of the gene hnRNPA2B1 exhibited a wrinkled pattern in 10 pairs of AGA hair follicle tissues as opposed to the normal hair follicle tissues (Figure 4B,C). The mRNA and protein levels of hnRNPA2B1 were increased in HFSCs from the normal hair follicle tissues as compared with HFSCs from the AGA hair follicle tissues (Figure 4D and Figure S3A), and hnRNPA2B1 could interact with and be pulled down from the AC010789.1 mRNA in HFSCs (Figure 4E). hnRNPA2B1 OE enhanced the mRNA levels of AC010789.1 as well as the expression levels of mRNA and protein of K6HF and Lgr5 in HFSCs (Figure 4F,G and Figure S3B), but AC010789.1 OE had no impact on the expression of hnRNPA2B1 in HFSCs (Figure S3C). hnRNPA2B1 OE increased the m^6^A levels of lncRNA AC010789.1 in HFSCs (Figure 4H), and AC010789.1 mRNAs could interact with and be enriched in hnRNPA2B1 protein in HFSCs (Figure 4I). Functionally, hnRNPA2B1 OE facilitated the proliferation, DNA synthesis and migration of HFSCs and AC010789.1 KD attenuated the promoting effects of hnRNPA2B1 OE on HFSCs growth (Figure 4J–L).

### 3.5 S100A8 was identified as a downstream regulator of AC010789.1 to enhance HFSCs growth by activating Wnt/β‐catenin signalling

**FIGURE 5:**
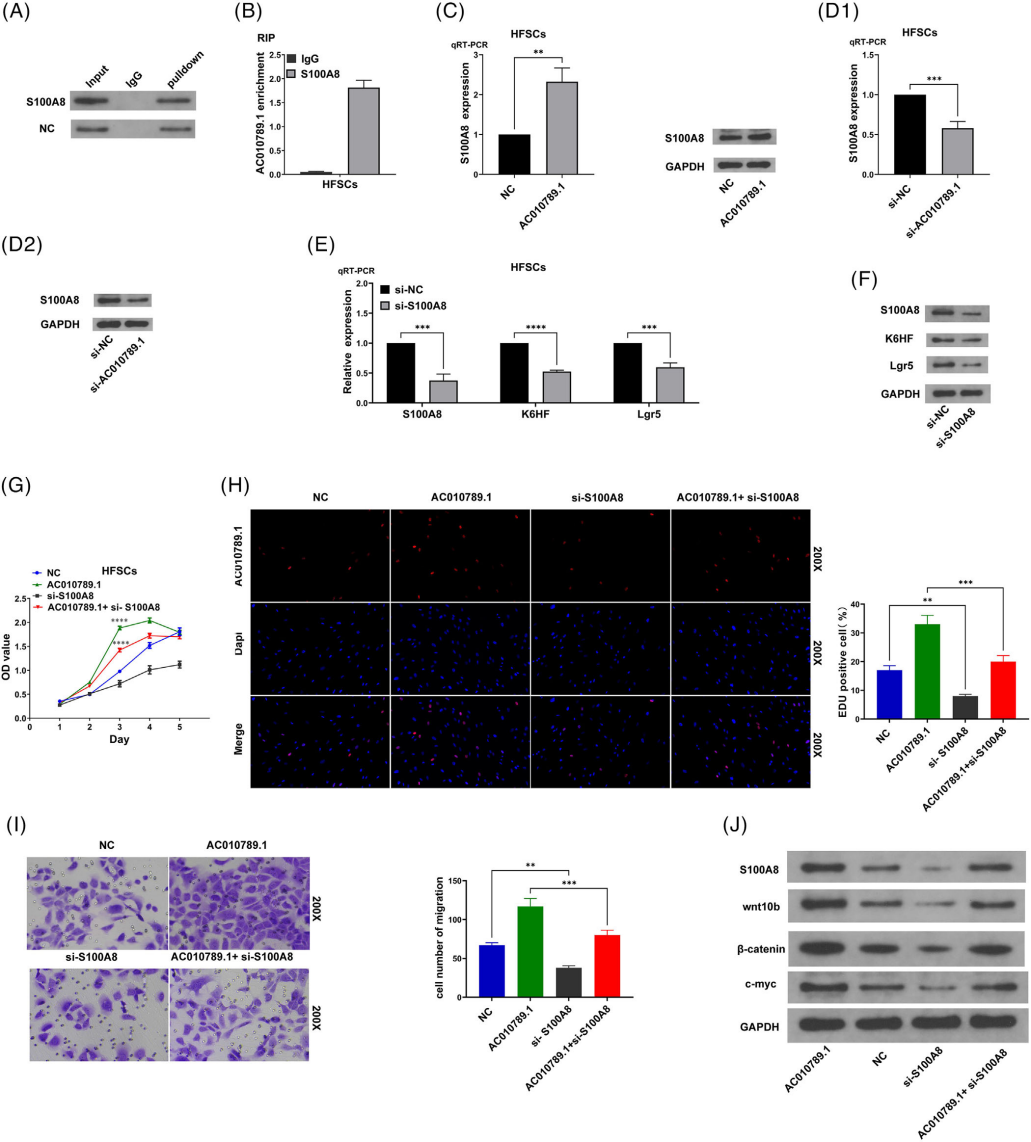
S100A8 was identified as a downstream regulator of AC010789.1 to promote hair follicle stem cells (HFSCs) growth by activating Wnt/β‐catenin signalling (A) RNA pull‐down analysis of the interaction of S100A8 protein with the AC010789.1 mRNAs in HFSCs. (B) RIP analysis of the endogenous enrichment of AC010789.1 mRNAs in S100A8 protein in HFSCs. (C, D) Real‐time quantitative polymerase chain reaction (qRT‐PCR) and Western blot analysis of the expression levels of S100A8 after the transfection with AC010789.1 OE lentiviruses or si‐AC010789.1 into HFSCs. (E, F) qRT‐PCR and Western blot analysis of the expression levels of S100A8, K6HF and Lgr5 after the transfection of si‐AC010789.1 into HFSCs. (G‐I) CCK8, 5‐ethynyl‐2'‐deoxyuridine assay (EdU) and Transwell analysis of the cell proliferation and migration viability after the co‐transfection with AC010789.1 OE lentiviruses and si‐S100A8 into HFSCs. (J) Western blot analysis of the expression levels of S100A8, Wnt10b, β‐catenin and c‐myc after the co‐transfection with AC010789.1 OE lentiviruses and si‐S100A8 into HFSCs. Data shown are the mean ± SEM of three experiments. **p < .01, ***p < .001 and ****p < .0001.

We found that lncRNA AC010789.1 mRNA also could bind with S100A8 protein in HFSCs by Mass Spectrum (Figure 4A). RNA pull‐down assay confirmed that S100A8 protein could bind with and be pulled down from the AC010789.1 mRNA in HFSCs (Figure 5A). RIP assay indicated AC010789.1 mRNA could interact with and be enriched in S100A8 protein in HFSCs (Figure 5B). quantitative real‐time PCR and Western blot revealed that AC010789.1 OE increased the expression levels of mRNA and protein of S100A8, whereas AC010789.1 KD decreased those of S100A8 in HFSCs (Figure 5C,D and Figure S4A). S100A8 KD reduced the expression levels of mRNA and protein of K6HF and Lgr5 in HFSCs (Figure 5E,F and Figure S4B) but had no impact on the level of AC010789.1 (Figure S4C). Functionally, S100A8 KD repressed the proliferation, DNA synthesis and migration of HFSCs and reversed the promoting effects of AC010789.1 OE on HFSCs growth (Figure 5G–I). Moreover, Western blot analyses indicated that AC010789.1 OE upregulated S100A8, Wnt10b, β‐catenin and c‐myc, but S100A8 KD reduced their protein levels and counteracted AC010789.1 OE‐induced their upregulation in HFSCs (Figure 5J and Figure S4D).

### 3.6 Exosome‐derived AC010789.1 accelerated HFSCs proliferation through the Wnt/β‐catenin signalling

**FIGURE 6:**
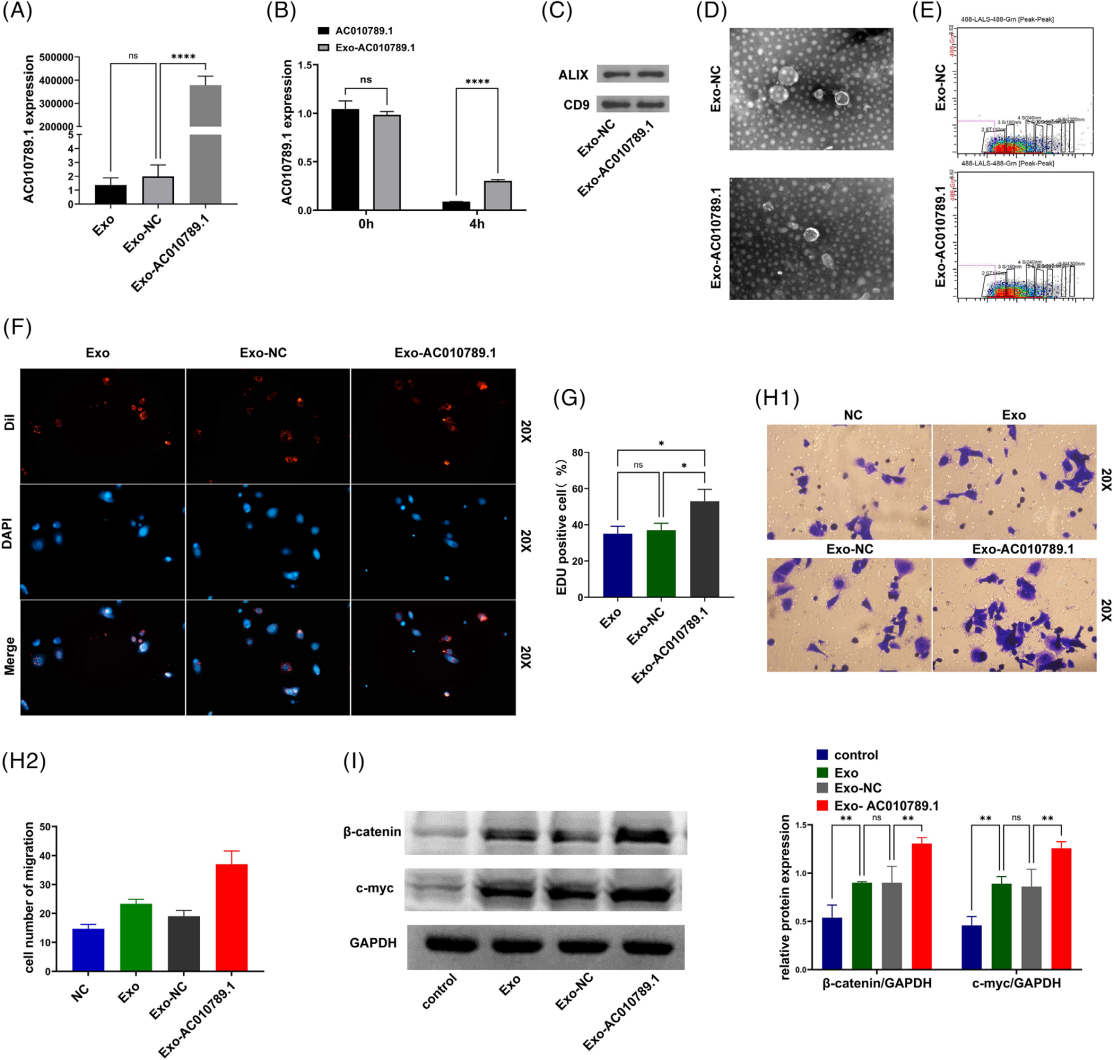
Exo‐AC010789.1 facilitated hair follicle stem cells (HFSCs) proliferation through the Wnt/β‐catenin signalling. (A) Real‐time quantitative polymerase chain reaction (qRT‐PCR) analysis of the expression levels of AC010789.1 in extracellular vesicles Exo‐AC010789.1 and Exo‐NC. (B) qRT‐PCR analysis of the stability of Exo‐AC010789.1. (C) Western blot analysis of the expression levels of extracellular vesicle markers ALIX and CD9 in Exo‐AC010789.1. (D) Microflow analysis of extracellular vesicle particle size. (E) Fluorescence microscopy of HFSCs absorbing exosomes. (F–H) 5‐ethynyl‐2'‐deoxyuridine assay (EdU), CCK8 and Transwell analysis of the effects of Exo‐AC010789.1 on HFSCs proliferation and migration. (I) Western blot analysis of the effects of Exo‐AC010789.1 on Wnt signalling transduction. Data shown are the mean ± SEM of three experiments. ns *p* ≥ .05,*p < .05, **p < .01 and ****p < .0001.

After the transfection with the AC010789.1 OE lentiviruses into ADSC, ADSC exosomes were extracted. qRT‐PCR verified a remarkable increase in AC010789.1 mRNA levels in the Exo‐AC010789.1 group in comparison to the Exo‐NC group (Figure 6A). After the exposure to actinomycin D for 4 h, quantitative real‐time PCR analysis displayed the increased stability of AC010789.1 in the Exo‐AC010789.1 group relative to the AC010789.1 group (Figure 6B). Western blot analysis indicated similar results for extracellular vesicle markers ALIX and CD9 between Exo‐AC010789.1 and Exo‐NC groups (Figure 6C). Exo‐AC010789.1 exhibited no significant difference in morphology from the normal exosomes by electron microscopy (Figure 6D). Microflow analysis also showed that Exo‐AC010789.1 harboured no significant difference in the size of extracellular vesicles from the normal exosomes (Figure 6E). We further utilized the extracellular vesicles to incubate with HFSCs. Exosome tracing experiment revealed that HFSCs could absorb Exo AC010789.1, and Exo‐AC010789.1 promoted HFSCs proliferation (Figure 6F,G) and migration (Figure 6H). Western blot analysis showed that the protein expression of β‐catenin and c‐myc were elevated by Exo‐AC010789.1 in comparison to the Exo‐NC group (Figure 6I).

### 3.7 Exosome‐derived AC010789.1 promoted hair regeneration in hairless mice

**FIGURE 7:**
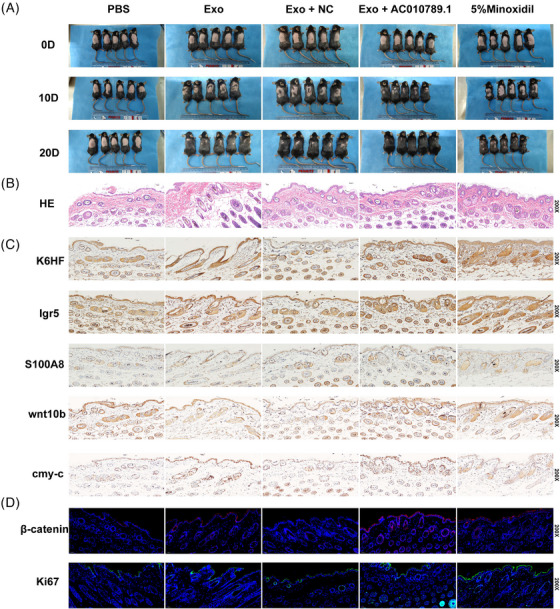
Exo AC010789.1 enhanced hair growth in hairless mice. (A) Observations of the area covered by hair on the back of mice. (B) Hematoxylin and Eosin (HE) analysis of skin tissue morphology (×200, scale 50µm). (C) Immunohistochemistry (IHC) analysis of the protein expression of K6HF, Lgr5, S100A8, Wnt10b and c‐myc. (D) IF detection of β‐catenin and Ki67 protein expression in skin tissues (×200, scale 50µm).

To validate the impact of Exo‐AC010789.1 on the hair growth of mice, we prepared a hairless mouse model using hair removal cream and subcutaneously injected the mouse back using exosomes Exo‐AC010789.1 and Exo‐NC each day. Local application of minoxidil was regarded as a positive control. The results showed that Exo‐AC010789.1 promoted hair growth in mouse hair follicles in comparison to the other groups (Figure 7A). HE analysis showed that Exo‐ AC010789.1 elevated the quantity of hair follicles on the back of mice and enhanced the growth of hair inside the follicles (Figure 7B). IHC analysis further demonstrated that the protein levels of K6HF, Lgr5, S100A8, Wnt10b and c‐myc were increased in the hair follicle tissues by Exo‐AC010789.1 as compared with the other groups (Figure 7C) and IF analysis verified the similar results for β‐catenin and Ki‐67 protein in Exo‐AC010789.1 group (Figure 7D).

## 4 DISCUSSION

HFSCs can propel the cyclic proliferation of hair follicles and offer novel insights into the treatment of AGA.^23^ PKM2 facilitates HFSCs proliferation and hair regeneration by upregulation of Wnt/β‐catenin signalling,^24^ BRD4 reduces HFSCs apoptosis in alopecia^25^ and ACER1 maintains the stabilized balance of HFSCs and the architecture of hair follicles.^26^ We previously identified lncRNA AC010789.1 upregulation in HFSCs.^13^ The cells have reached confluence by the fourth day, and subsequently, they begin to undergo a gradual decline in viability. Despite this interesting observation, we can still confirm that lncRNA AC010789.1 was upregulated in HFSCs and accelerated HFSCs proliferation and migration, indicating that lncRNA AC010789.1 could be used for the treatment of AGA.

The m^6^A RNA methylation plays a critical part in various human ailments, with particular relevance to dermatological conditions.^14^, ^15^, ^16^ Tumour necrosis factor‐α Inhibits the developmental progression of mesenchymal stromal cells into sweat glands by blocking FTO‐mediated m6A methylation of Nanog mRNA,^27^ METTL14‐mediated m^6^A modification of Col17a1 and integrin β4/α6 determines epidermal homeostasis,^28^ and METTL3‐mediated m^6^A modification of ΔNp63 promotes cutaneous squamous cell carcinoma.^29^ Moreover, m^6^A methylation of lncRNA Pvt1 can govern skin tissue homeostasis.^30^ We herein found that FTO was downregulated in HFSCs and inhibition of FTO increased AC010789.1 m^6^A levels and its mRNA levels in HFSCs. FTO inhibition‐mediated m^6^A modification of AC010789.1 facilitated HFSCs proliferation. Moreover, it has been verified that hnRNPA2B1 regulates endocrine resistance^31^ and mediates ATG4B decay to enhance olaparib resistance in breast cancer.^32^ We further found that lncRNA AC010789.1 could interact with hnRNPA2B1, which was downregulated in AGA but upregulated in HFSCs, and hnRNPA2B1‐mediated m^6^A modification of AC010789.1 promoted HFSCs proliferation. These results indicated that m^6^A modification of AC010789.1 facilitated HFSCs proliferation.

S100A8 has been shown to act in skin diseases.^33^ S100A8 displays the association with disease activity of psoriasis^34^ and skin cancer,^35^ and S100A8 deficiency prompts skin hyperplasia.^36^ Moreover, S100A8 can induce the activation of Wnt/β‐catenin pathway implicated in osteoarthritis^37^ and colorectal cancer.^38^ In our study, we found that lncRNA AC010789.1 could interact with the downstream S100A8 protein, activate Wnt/β‐catenin signalling and accelerate HFSCs proliferation. These results indicated that m^6^A modification of AC010789.1 activated S100A8‐mediated Wnt/β‐catenin signalling to promote HFSCs proliferation.

Exosomes have been applied for various disease therapeutics.^39^ Exosomes‐derived lncRNA DARS‐AS1 siRNA can repress the metastasis of triple‐negative breast cancer^40^ and exosome of lncRNA LINC01140 hinders breast tumor development by inactivating Wnt/β‐Catenin signalling.^41^ Also, exosomes from the hair papilla cells can be utilized for the treatment of AGA^42^ and adipose‐derived stem cell exosomes can enhance hair follicle development through the stimulation of Wnt/β‐Catenin signalling.^43^ We herein confirmed that exosome‐loaded lncRNA AC010789.1 in HFSCs could promote hair follicle regeneration in mice and provide a novel therapeutic strategy for AGA.

## 5 CONCLUSIONS

**FIGURE 8:**
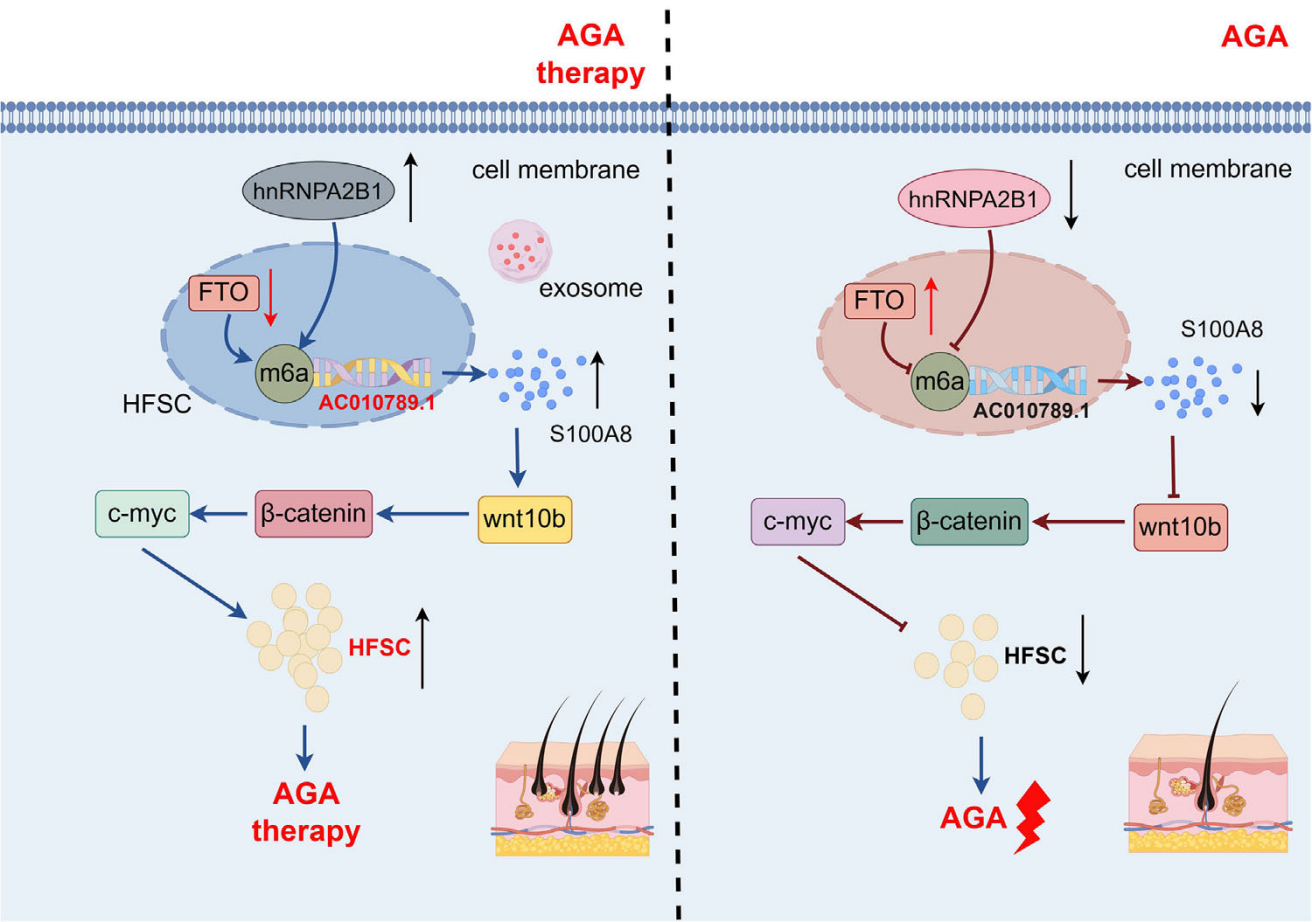
Schematic diagram of the molecular mechanism of long non‐coding RNA (lncRNA) AC010789.1 in hair follicle stem cells (HFSCs) against androgenic alopecia (AGA). FTO/hnRNPA2B1 mediates m^6^A modification of lncRNA AC010789.1 to activate S100A8/Wnt/β‐catenin signalling, contributing to HFSCs growth and exosome‐derived AC010789.1 in HFSCs could be applied for treatment of AGA.

To summarize, our findings demonstrated that FTO/hnRNPA2B1‐mediated m^6^A modification of lncRNA AC010789.1 promoted HFSCs growth by activating S100A8/Wnt/β‐catenin signalling and exosome‐derived AC010789.1 in HFSCs could be applied for the treatment of AGA (Figure 8).

## AUTHOR CONTRIBUTIONS

Shaojun Chu conceived and designed the experiments; Shaojun Chu, Lingling Jia, Yulong Li, Jiachao Xiong, Yulin Sun, Qin Zhou and Dexiang Du performed the experiments; Shaojun Chu, Zihan Li and Xin Huang analyzed the data; Shaojun Chu, Hua Jiang, Baojin Wu and Yufei Li wrote the paper. All authors read and approved the final manuscript.

## CONFLICT OF INTEREST STATEMENT

The authors declare no conflict of interest.

## ETHICS STATEMENT

Ethical approval was obtained from Shanghai East Hospital and conducted in accordance with the principles of the Declaration of Helsinki and the Basel Declaration. The present study was approved by the Ethics Committee of Shanghai East Hospital (No. 2021‐KYSB‐148, 2021‐2‐26).

## Supporting information



Supporting Information

Supporting Information

Supporting Information

Supporting Information

Supporting Information

## Data Availability

All data generated or analyzed in this study are included in this article and its supplementary information files.
