# Supplementary material for: Exosome‐derived long non‐coding RNA AC010789.1 modified by FTO and hnRNPA2B1 accelerates growth of hair follicle stem cells against androgen alopecia by activating S100A8/Wnt/β‐catenin signalling
Source: Clin Transl Med. 2025 Jan 2;15(1):e70152. doi: 10.1002/ctm2.70152 (PMC11695201; doi:10.1002/ctm2.70152)
Supplement: Supplementary file 1 — Supporting Information [file CTM2-15-e70152-s004.docx]

**Supplementary Figure legends**

**Figure S1.** Western blot analysis of the protein levels of K6HF and Lgr5 after the transfection with AC010789.1 OE lentiviruses or si-AC010789.1 into HFSCs. (**A**) Western blot analysis of the expression levels of K6HF and Lgr5 after the transfection with AC010789.1 OE lentiviruses into HFSCs. (**B**) Western blot analysis of the expression levels of K6HF and Lgr5 after transfection with si-AC010789.1 into HFSCs. Data shown are the mean ± SEM of three experiments. *****P* < 0.0001.

**Figure S2.** qRT-PCR and Western blot analysis of the expression levels of AC010789.1, FTO, K6HF and Lgr5 in HFSCs. (**A**) Analysis of the m^6^A modification sites of AC010789.1. (**B**) Western blot analysis of the expression levels of FTO in HFSCs as compared with the normal follicle cells. (**C**) Western blot analysis of the expression levels of FTO, K6HF and Lgr5 after the transfection with FTO into HFSCs. (**D, E**) qRT-PCR analysis of the expression levels of ALBKH5 and AC010789.1 after the transfection with ALBKH5 into HFSCs. (**F, G**) qPRT-CR analysis of the expression levels of METTL3 and AC010789.1 after the transfection with METTL3 into HFSCs. Data shown are the mean ± SEM of three experiments. **P* < 0.05, ***P* < 0.01, ****P* < 0.001.

**Figure S3.** Western blot analysis of the protein levels of hnRNPA2B1, K6HF and Lgr5 after the transfection with hnRNPA2B1 OE plasmids into HFSCs. (**A**) Western blot analysis of the expression levels of hnRNPA2B1 in HFSCs as compared with the normal follicle cells. (**B**) Western blot analysis of the expression levels of hnRNPA2B1, K6HF and Lgr5 after the transfection with hnRNPA2B1 plasmids into HFSCs. (**C**) qRT-PCR analysis of the expression levels of hnRNPA2B1 after the transfection with AC010789.1 OE lentiviruses into HFSCs. Data shown are the mean ± SEM of three experiments. ***P* < 0.01, ****P* < 0.001.

**Figure S4.** Western blot analysis of the effects of S100A8 on the protein levels of K6HF and Lgr5 in HFSCs. (**A**) Western blot analysis of the expression levels of S100A8 after the transfection with AC010789.1 OE lentiviruses or si-AC010789.1 into HFSCs. (**B**) Western blot analysis of the expression levels of S100A8, K6HF and Lgr5 after transfection with si-S100A8 into HFSCs. (**C**) RT-qPCR analysis of the expression levels of AC010789.1 after the transfection with si-S100A8 into HFSCs. (**D**) Western blot analysis of the expression levels of S100A8, Wnt10b, β-catenin and c-myc after co-transfection with AC010789.1 OE lentiviruses and si-S100A8 into HFSCs. Data shown are the mean ± SEM of three experiments. ****P* < 0.001, *****P* < 0.0001.

**Table S1** Sequences of primers in the study

| Name | Primer | Sequence | Size |  |
| --- | --- | --- | --- | --- |
| Homo β-actin | Forward | CCCTGGAGAAGAGCTACGAG | 180bp |  |
|  | Reverse | CGTACAGGTCTTTGCGGATG |  |  |
| Homo FTO | Forward | CGGTATCTCGCATCCTCATT | 200bp |  |
|  | Reverse | GGCAGCAAGTTCTTCCAAAG |  |  |
| Homo AC010789.1 | Forward | TGCATCCCTGGCAATACTCAG |  |  |
|  | Reverse | GGAGTGCTGTGCATTCATTGG |  |  |
| Homo K6HF | Forward | TTTCTGTGGTCACCTCTACTCT | 216bp |  |
|  | Reverse | AGGATGTGGTGGAGACAAACTT |  |  |
| Homo lgr5 | Forward | CTCTTCCTCAAACCGTCTGC | 181bp |  |
|  | Reverse | GATCGGAGGCTAAGCAACTG |  |  |
| Homo ALKBH5 | Forward | CAAAGTCCCTGAGCAGCTCCGTGTT | 167bp |  |
|  | Reverse | GCGCCGGTTCTCTTCCTTGTCCATC |  |  |
| Homo METTL3 | | Forward | AACCTCTGGGGGTATGAACGGGTA | 151bp |
|  |  | Reverse | TGAAGCCTTGGGGATTTCCTTTGAC |  |
| Homo HNRNPA2B1 | | Forward | TAATGAGGGATCCTGCAAGC | 177bp |
|  |  | Reverse | CAGTTACATGAGCCCCTGGT |  |
| Homo S100A8 | | Forward | ATGCCGTCTACAGGGATGAC | 193bp |
|  |  | Reverse | TGGCTTTCTTCATGGCTTTT |  |
