# Supplementary figures and images for: Exosome‐derived long non‐coding RNA AC010789.1 modified by FTO and hnRNPA2B1 accelerates growth of hair follicle stem cells against androgen alopecia by activating S100A8/Wnt/β‐catenin signalling
Source: Clin Transl Med. 2025 Jan 2;15(1):e70152. doi: 10.1002/ctm2.70152 (PMC11695201; doi:10.1002/ctm2.70152)

**A**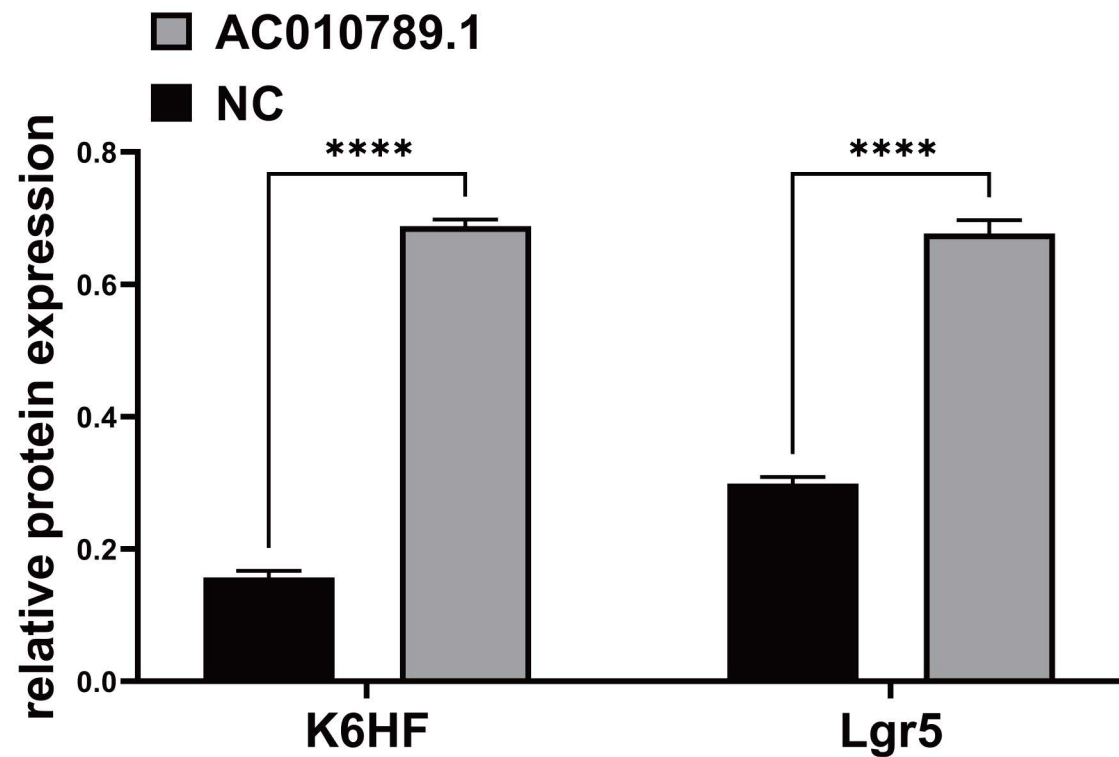**B**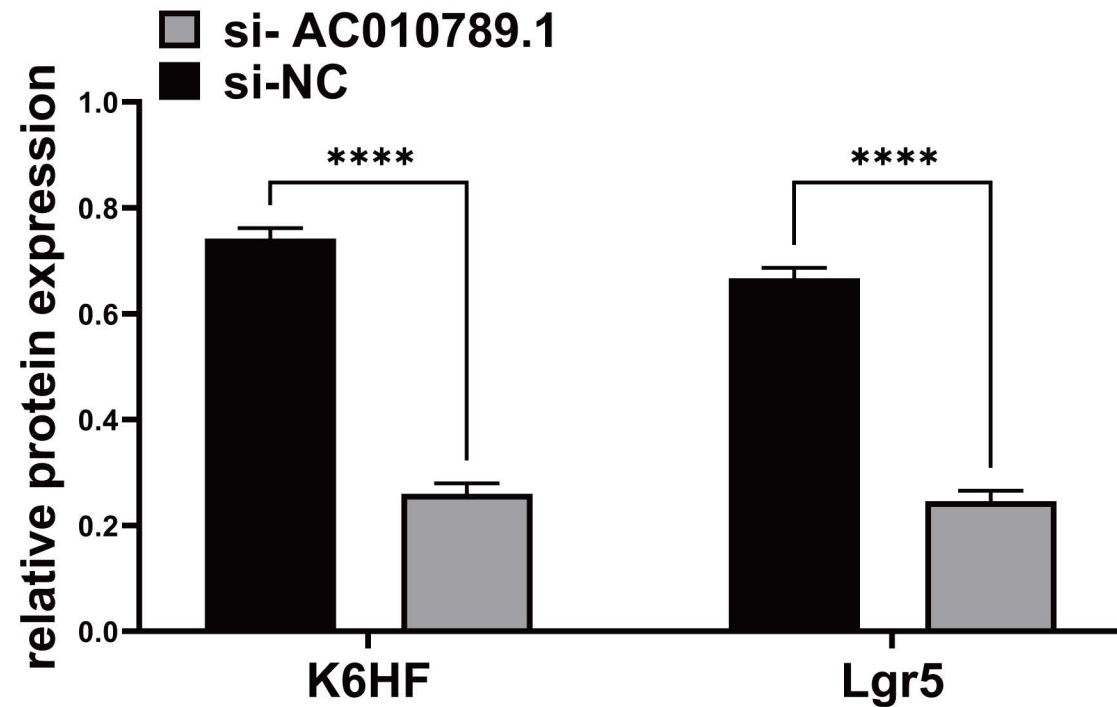

Supplement: Supplementary file 2 — Supporting Information [file CTM2-15-e70152-s002.pdf]

**A**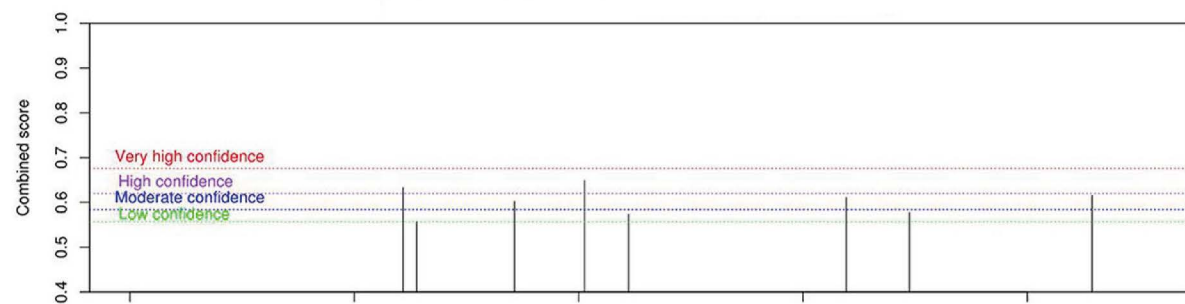**B**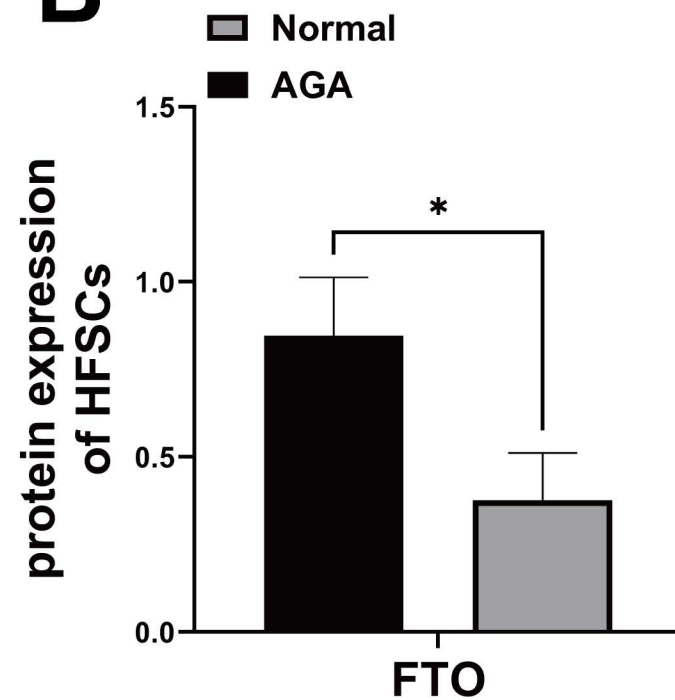**C**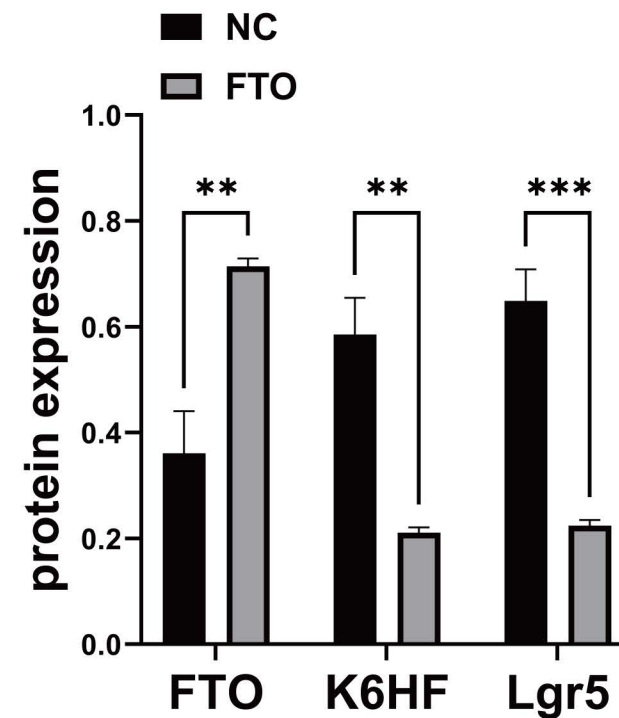**D**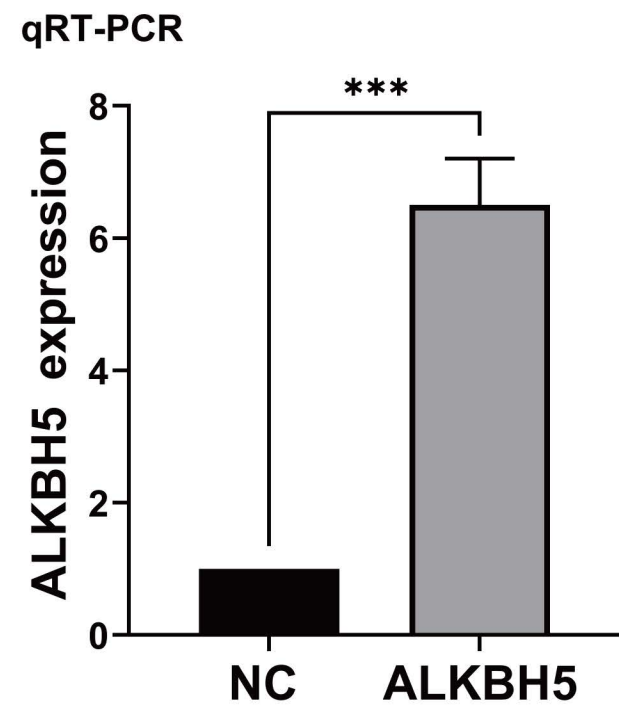**E**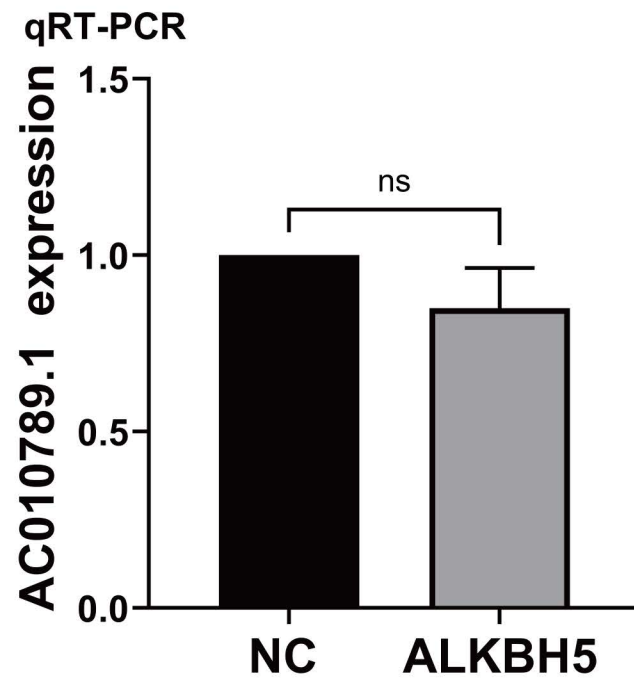**F**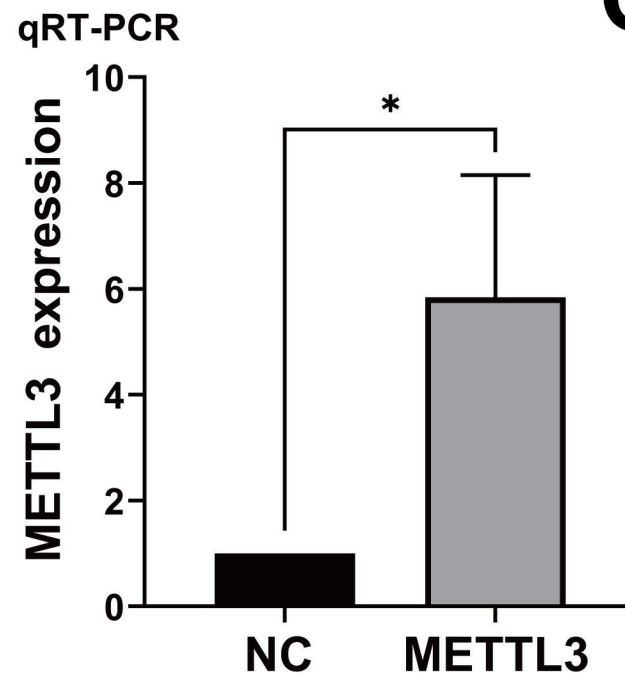**G**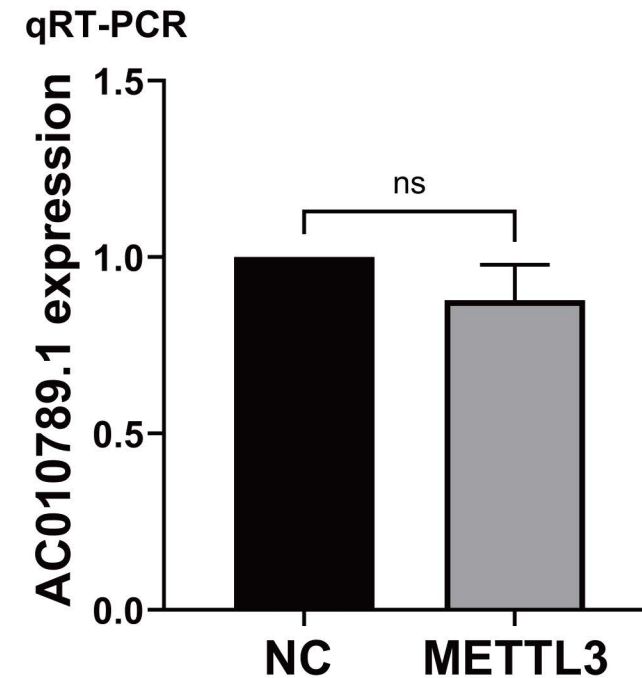

Supplement: Supplementary file 3 — Supporting Information [file CTM2-15-e70152-s003.pdf]

**A**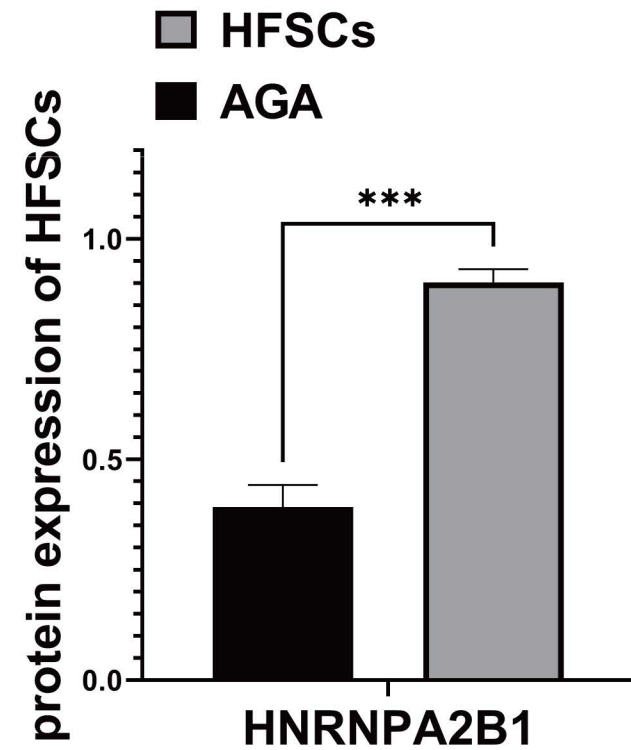**B**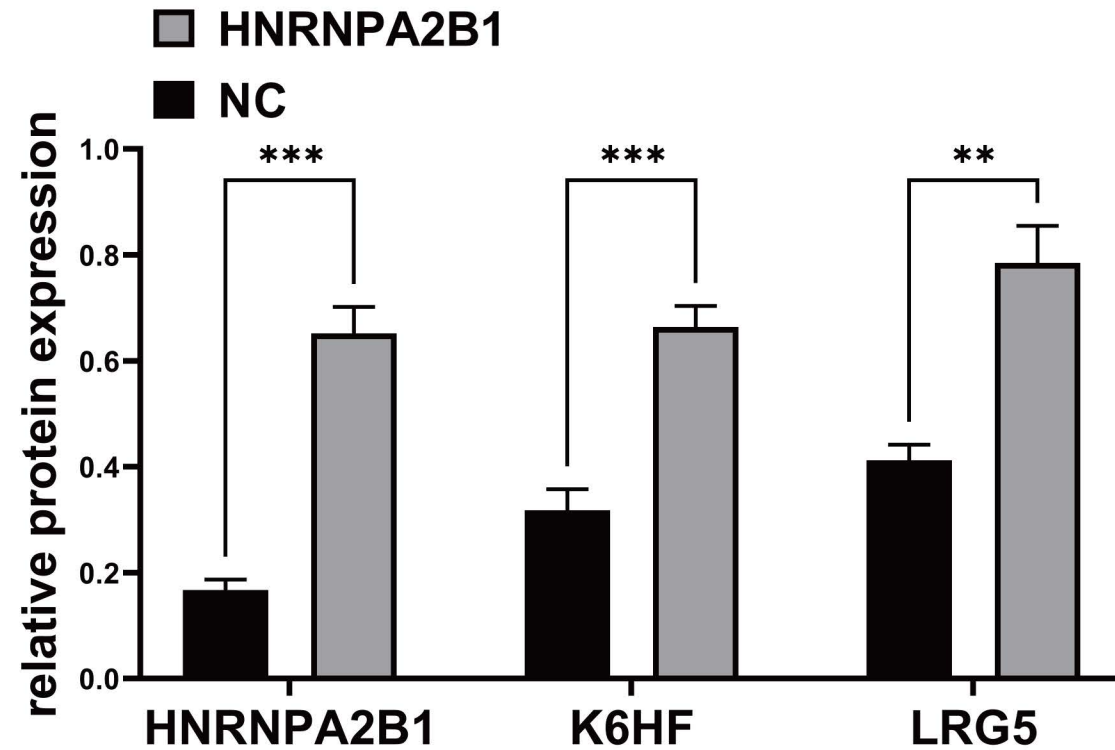**C**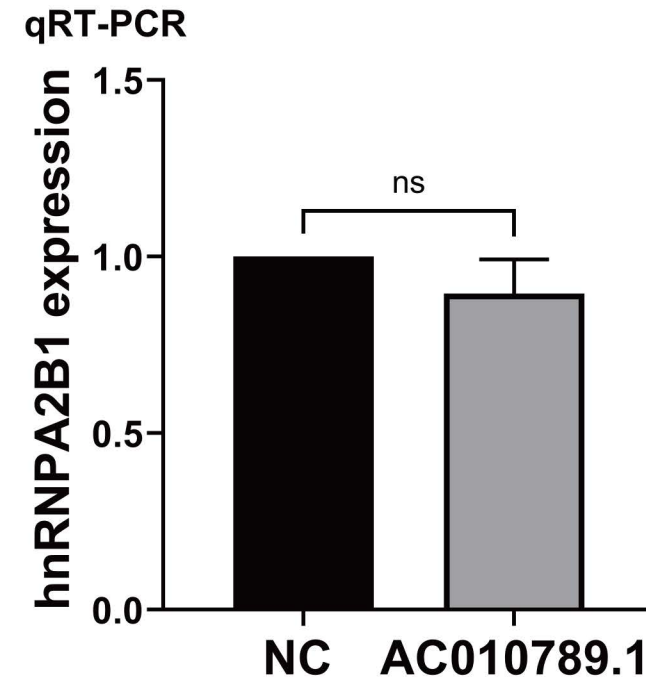

Supplement: Supplementary file 4 — Supporting Information [file CTM2-15-e70152-s001.pdf]

**A**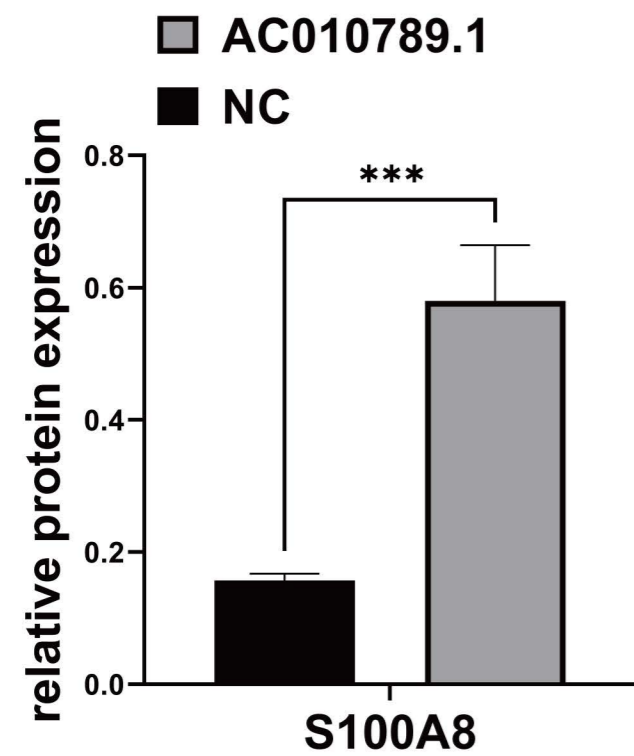**B**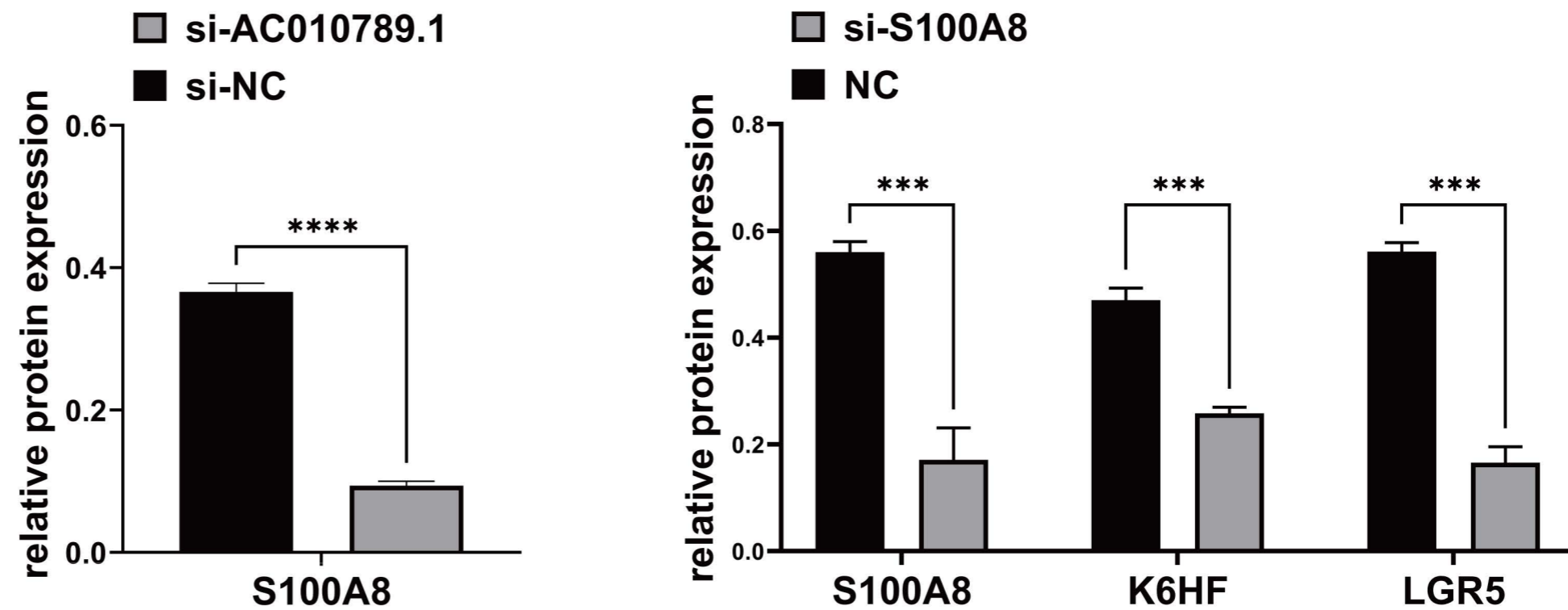**C**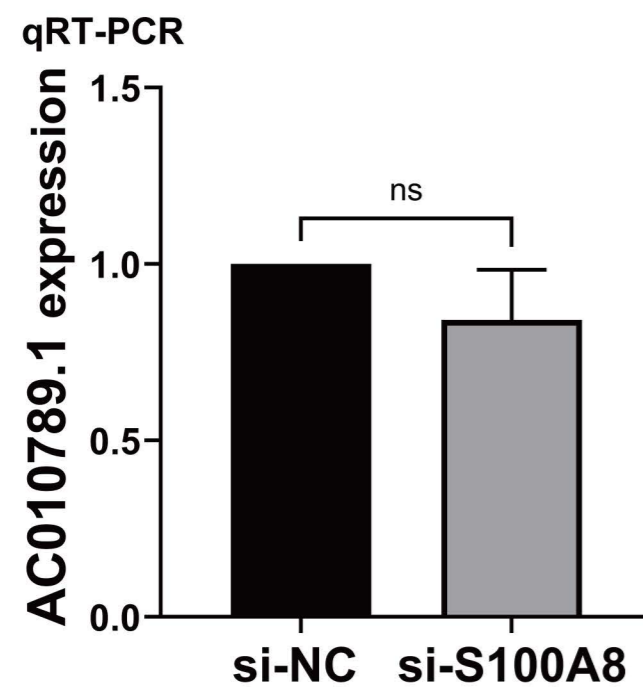**D**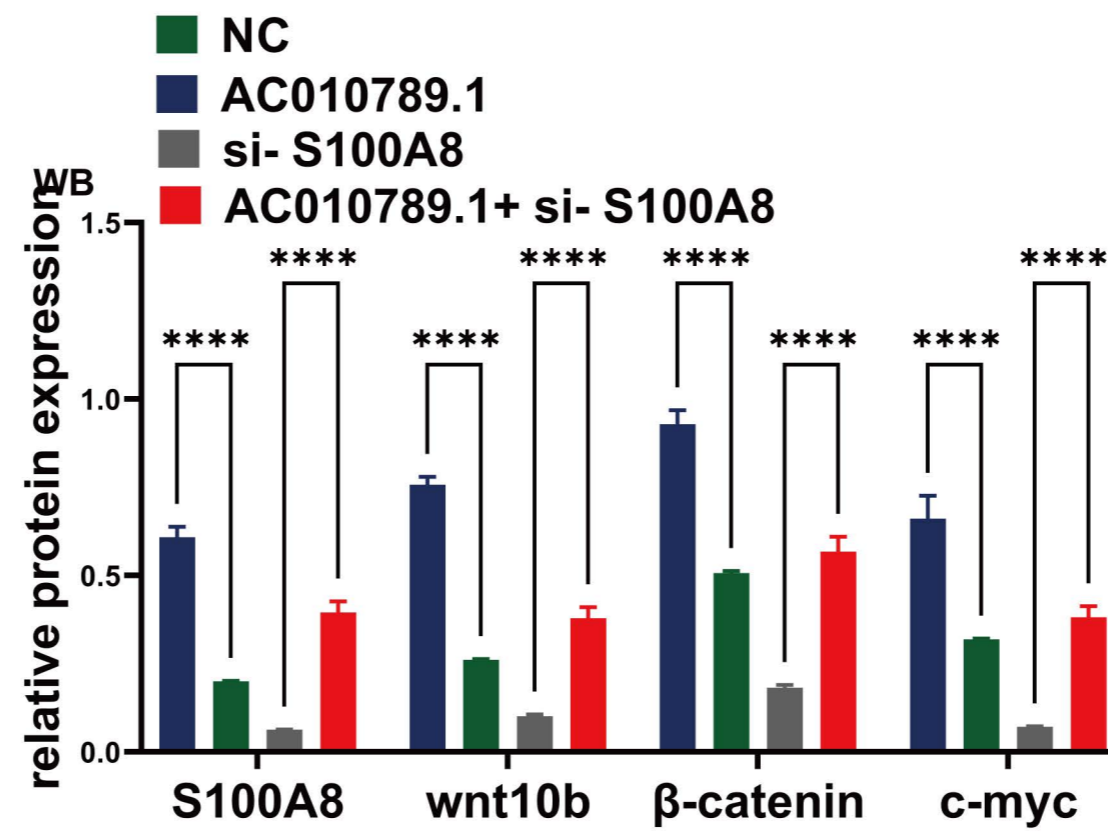

Supplement: Supplementary file 5 — Supporting Information [file CTM2-15-e70152-s005.pdf]
